# Evaluation of the Immediate Effects of Web-Based Intervention Modules for Goals, Planning, and Coping Planning on Physical Activity: Secondary Analysis of a Randomized Controlled Trial on Weight Loss Maintenance

**DOI:** 10.2196/35614

**Published:** 2022-04-14

**Authors:** Elina Mattila, Graham Horgan, António L Palmeira, Ruairi O'Driscoll, R James Stubbs, Berit L Heitmann, Marta M Marques

**Affiliations:** 1 VTT Technical Research Centre of Finland Ltd Tampere Finland; 2 Biomathematics and Statistics Scotland Edinburgh United Kingdom; 3 Centro de Investigação em Desporto, Educação Física, Exercício e Saúde Faculdade de Educação Física e Desporto Universidade Lusófona de Humanidades e Tecnologias Lisbon Portugal; 4 School of Psychology Faculty of Medicine and Health University of Leeds Leeds United Kingdom; 5 Research Unit for Dietary Studies The Parker Institute Bispebjerg and Frederiksberg Hospital Copenhagen Denmark; 6 Department of Public Health Section for General Practice University of Copenhagen Copenhagen Denmark; 7 Comprehensive Health Research Centre NOVA Medical School Universidade Nova de Lisboa Lisbon Portugal

**Keywords:** digital intervention, Fitbit, weight, weight loss maintenance, physical activity, fitness, exercise, goal setting, action planning, coping planning, control trial, secondary analysis, RCT, randomized controlled trial, long-term effect, short-term effect, immediate effect, sustained effect

## Abstract

**Background:**

The use of digital interventions can be accurately monitored via log files. However, monitoring engagement with intervention goals or enactment of the actual behaviors targeted by the intervention is more difficult and is usually evaluated based on pre-post measurements in a controlled trial.

**Objective:**

The objective of this paper is to evaluate if engaging with 2 digital intervention modules focusing on (1) physical activity goals and action plans and (2) coping with barriers has immediate effects on the actual physical activity behavior.

**Methods:**

The NoHoW Toolkit (TK), a digital intervention developed to support long-term weight loss maintenance, was evaluated in a 2 x 2 factorial randomized controlled trial. The TK contained various modules based on behavioral self-regulation and motivation theories, as well as contextual emotion regulation approaches, and involved continuous tracking of weight and physical activity through connected commercial devices (Fitbit Aria and Charge 2). Of the 4 trial arms, 2 had access to 2 modules directly targeting physical activity: a module for goal setting and action planning (Goal) and a module for identifying barriers and coping planning (Barriers). Module visits and completion were determined based on TK log files and time spent in the module web page. Seven physical activity metrics (steps; activity; energy expenditure; fairly active, very active and total active minutes; and distance) were compared before and after visiting and completing the modules to examine whether the modules had immediate or sustained effects on physical activity. Immediate effect was determined based on 7-day windows before and after the visit, and sustained effects were evaluated for 1 to 8 weeks after module completion.

**Results:**

Out of the 811 participants, 498 (61.4%) visited the Goal module and 406 (50.1%) visited the Barriers module. The Barriers module had an immediate effect on very active and total active minutes (very active minutes: before median 24.2, IQR 10.4-43.0 vs after median 24.9, IQR 10.0-46.3; *P*=.047; total active minutes: before median 45.1, IQR 22.9-74.9 vs after median 46.9, IQR 22.4-78.4; *P*=.03). The differences were larger when only completed Barriers modules were considered. The Barriers module completion was also associated with sustained effects in fairly active and total active minutes for most of the 8 weeks following module completion and for 3 weeks in very active minutes.

**Conclusions:**

The Barriers module had small, significant, immediate, and sustained effects on active minutes measured by a wrist-worn activity tracker. Future interventions should pay attention to assessing barriers and planning coping mechanisms to overcome them.

**Trial Registration:**

ISRCTN Registry ISRCTN88405328; https://www.isrctn.com/ISRCTN88405328

## Introduction

Digital behavior change interventions (DBCIs) hold the promise of providing personalized and adaptive treatments to improve health. It is already possible to track individual user interactions with DBCI components at a high level of detail and fidelity, such as when and for how long users are accessing them. The evidence linking use to intervention outcomes is mixed, and the relationships are not straightforward. Further, the type of engagement required for effects may also vary across different types of interventions [1,2].

User engagement with the real-world behaviors that a DBCI is attempting to influence, the macro-level engagement [1], is more difficult to determine. Traditionally, behavioral changes are verified using questionnaires or laboratory measurements conducted intermittently, with measurement points several months apart and the risk of reporting bias. At that point, it is impossible to prove causation between specific intervention components and change. Monitoring the immediate effects of DBCI components would enable active process evaluation of interventions and contribute greatly to tailoring effective, adaptive, and personalized interventions. For example, if an intervention component has an immediate effect on the user’s behavior, the component could be repeated after a while to amplify the effect. If a component does not lead to an expected effect for a user, it could be switched off and an alternative intervention could be launched to create an adaptive intervention [3].

Research on behavior change techniques has revealed the positive effects of self-regulatory techniques on physical activity behavior. Core self-regulatory techniques are self-monitoring of behavior and feedback; goal setting, that is, identification and formulation of a physical activity goal; action planning, that is, specification of the goal in a detailed plan for the performance of the behavior (context, frequency, duration, and the intensity of the activity); and coping planning, that is, identification of barriers to physical activity and planning ways to overcome them using, for example, an “if, then” approach [4-6].

This study focuses on a web-based toolkit for weight loss maintenance, consisting of various modules based on behavioral self-regulation and motivation theories, as well as contextual emotion regulation. The objective of this study was to investigate whether intervention modules aimed at increasing physical activity through goal setting and action planning, and coping planning had immediate and sustained effects on the physical activity behavior of users. This was a secondary analysis of the data from a European Commission Horizon 2020–funded NoHoW project.

## Methods

### Ethics Approval

The trial was registered with the ISRCTN registry (ISRCTN88405328). Ethical approval was granted by local institutional ethics committees at the Universities of Leeds (17-0082; 27 February 2017), Lisbon (17/2016; 20 February 2017) and the Capital Region of Denmark (H-16030495; 8 March 2017).

### Study Procedures

The NoHoW trial (ISRCTN88405328) was an 18-month, 3-center, 2-by-2 factorial, single-blinded, randomized controlled trial, evaluating a digital weight loss maintenance intervention. The participants were required to be aged ≥18 years, have a verified ≥5% weight loss in the last 12 months with current weight at least 5% below their highest weight, and have had a BMI of ≥25 kg/m^2^ before weight loss. A total of 1627 participants were recruited and randomly assigned to one of the following four arms: (1) control or self-monitoring (n=400), (2) *motivation and self-regulation* (n=403), (3) *emotion regulation* (n=416), and (4) *combined* arm (n=408). All participants received activity trackers (Fitbit Charge 2) to be worn throughout the trial, weight scales (Fitbit Aria), and access to the web-based NoHoW Toolkit (TK) tailored to their respective arm. Participants in intervention arms were encouraged to complete 18 intervention modules in the TK during the first 6 months of the trial. The participants received weekly emails during the first 18 weeks as reminders recommending visiting a specific module. A detailed description of the trial is presented by Scott et al [7]. The TK design and content are presented in detail by Marques et al [8].The *motivation and self-regulation* and *combined* arms had 2 modules focusing on physical activity: physical activity goal (Goal) and physical activity barriers (Barriers). The Goal module (see Multimedia Appendix 1 for screenshots) addressed goal setting and action planning, contained information on how to set goals, and had a form to set a goal and detailed plan for either the number of steps per day or other type of physical activity. The estimated duration to complete the Goal module was 10 minutes. The Barriers module (see Multimedia Appendix 2 for screenshots) was introduced later in the intervention and contained a testimonial on potential barriers of physical activity and an interactive exercise for identifying personal barriers and creating a coping plan to deal with them. The estimated duration to complete the Barriers module was 8 minutes.

### Analysis

Visits to the Goal and Barriers modules were identified based on log files of the TK. The duration spent in the modules was calculated based on log events signifying entering and leaving the module. A module visit was considered complete if it lasted at least 33% of the estimated duration of the module (ie, 3.3/10 min for the Goal module and 2.6/8 min for the Barriers module) or if the duration of the visit could not be determined due to a missing end event. The threshold of 33% was determined by intervention designers as the minimum time required to become exposed to the behavior change mechanisms in these modules.

Daily summaries provided by Fitbit were used as the physical activity metrics and included daily steps; activity energy expenditure; active minutes categorized to fairly active, very active, and total active minutes; and distance. Activity metrics were averaged over the 7 days prior to visiting the modules and 7 days after visiting them. Days with less than 1000 steps were considered missing data and were not included. A similar threshold has been used in several previous studies (eg, [9,10]). It was also required that the 7-day periods contained at least 4 days of activity data. The immediate effects are presented both for all visits to the modules and for complete visits. If an immediate effect was found, maintenance of the effects was evaluated for 8 weeks following the module visits, considering only complete module visits. A comparison of overall changes in activity metrics between the first and sixth month of the study is presented in Multimedia Appendix 3.

As most of the physical activity metrics had skewed distributions, nonparametric methods were used. Median and interquartile ranges were calculated, and nonparametric tests (Wilcoxon signed-rank test) were used for comparisons. All analyses were conducted with Matlab R2017a (Mathworks) and SPSS Statistics software (version 26; IBM Corp). Statistical significance was set at *P*<.05.

## Results

### User Statistics

The modules were available for 811 participants (ie, the participants randomized to the *motivation and self-regulation* and to the *combined* arms). Of the 811 participants, 498 (61.4%) visited the Goal module (252/403 *motivation and self-regulation* and 246/408 *combined*), and 406 (50.1%) participants visited the Barriers module (217/403 *motivation and self-regulation* and 189/408 *combined*). There were 628 visits to the Goal module, of which 309 were complete visits. The Barriers module had 514 visits, with 345 complete. The background characteristics of the visitors and nonvisitors of these modules are presented and compared in Multimedia Appendix 3.

### Immediate Effects of Modules

The Goal module was first visited a median of 55 days (IQR 48-64) after the first login to TK, and the Barriers module was first visited a median of 98 days (IQR 91-110) after the first login. Tables 1 and 2 present the median and IQR for the activity metrics before and after visiting the Goal and Barriers modules, respectively. Visiting the Barriers module increased very active and total active minutes.

When only completed module visits were included, the results for the Goal module remained nonsignificant. For the Barriers module, the effects remained and became slightly stronger (for very active minutes: before median 22.4, IQR 10.1-41.0 vs after median 25.0, IQR 10.1-46.1; *P*=.007; and for total active minutes: before median 42.6, IQR 22.4-73.4 vs after median 46.6, IQR 21.8-79.4; *P*=.008).

**Table 1:** Activity metrics before and after visiting the Goal module.

	Before, median (IQR)	After, median (IQR)	*P* value^a^
Steps	9637 (7383-12,485)	9638 (7354-12,425)	.62
Energy (kcal)	1276 (997-1632)	1274 (987-1640)	.74
Fairly active (min)	18.9 (10.2-33.3)	18.3 (11.0-32.5)	.69
Very active (min)	23.9 (12.2-44.8)	26.4 (12.6-44.8)	.39
Total active (min)	46.6 (25.1-75.4)	48.3 (26.6-76.5)	.44
Distance (km)	6.8 (5.1-8.9)	6.8 (5.1-8.7)	.60

^a^Wilcoxon signed-rank test.

**Table 2:** Activity metrics before and after visiting the Barriers module.

	Before, median (IQR)	After, median (IQR)	*P* value^a^
Steps	9449 (7240-12,112)	9119 (7202-12,382)	.70
Energy (kcal)	1211 (990-1585)	1260 (943-1611)	.27
Fairly active (min)	16.7 (9.7-31.9)	18.9 (9.6-33.8)	.15
Very active (min)	24.2 (10.4-43.0)	24.9 (10.0-46.3)	.047
Total active (min)	45.1 (22.9-74.9)	46.9 (22.4-78.4)	.03
Distance (km)	6.7 (4.9-8.5)	6.4 (4.9-8.7)	.68

^a^Wilcoxon signed-rank test.

Table 3 presents the 8-week maintenance of the Barriers module effect for the 3 active minute metrics based on completed module visits only. Total active minutes and fairly active minutes were higher than before module completion for most of the 8-week period following the module. Also, very active minutes remained higher for 3 weeks after module completion. Values that significantly differed from the before value were denoted.

**Table 3:** Median (IQR) values for active minutes categories before and after the Barriers module based on completed modules.

	Fairly active (min), median (IQR)	Very active (min), median (IQR)	Total active (min), median (IQR)
Before	15.9 (9.4-29.4)	22.4 (10.1-41.0)	42.6 (22.4-73.4)
Week 1 after	18.4 (9.2-33.3)	25.0 (10.1-46.1)^a^	46.6 (21.8-79.4)^a^
Week 2 after	19.1 (10.9-31.8)^a^	26.6 (12.0-45.0)^b^	45.6 (26.4-75.7)^b^
Week 3 after	18.6 (10.2-34.6)^b^	24.4 (11.9-45.6)^a^	44.6 (25.3-78.2)^a^
Week 4 after	18.1 (11.6-37.0)^b^	23.1 (12.0-43.2)	47.2 (25.2-76.8)^a^
Week 5 after	19.6 (10.6-30.6)^a^	24.4 (11.9-42.1)	44.7 (25.7-72.3)
Week 6 after	19.6 (11.4-33.3)^b^	23.6 (12.4-41.2)	45.3 (27.1-78.5)^a^
Week 7 after	20.3 (11.2-32.7)^b^	24.0 (12.0-40.9)	44.4 (27.6-74.4)^c^
Week 8 after	20.3 (11.2-31.7)^a^	23.7 (11.2-42.3)	46.1 (26.7-73.4)

^a^*P*<.01, Wilcoxon signed-rank test.

^b^*P*<.001.

^c^*P*<.05.

## Discussion

### Principal Results

This paper investigates the immediate changes in measured physical activity after visiting 2 web-based intervention modules targeting physical activity goals and action planning (Goal), and coping planning (Barriers). The Barriers module had a significant but small, immediate effect on very active and total active minutes during the week after visiting the module. When only completed module visits were considered, increases were larger. Module completion was also associated with sustained increases in all categories of active minutes, of which fairly active minutes and total active minutes were sustained for most of the 8-week period following module completion, and very active minutes for the first 3 weeks. Coping planning, addressed in the Barriers module, has previously been identified as a core self-regulation technique that can directly impact behavior (eg, [11,12]), and our findings support this effect for physical activity. The Goal module did not show similar effects on physical activity. One explanation for the differences in the results is that the coping planning activity in the Barriers module asked participants to identify barriers and strategies to overcome them in the immediate future (following week), while in the Goal module participants were asked to set a goal to increase their activity moderately in an unspecified timeframe.

### Strengths and Limitations

The strengths of this study include the large number of participants and the ability to monitor the exposure to specific intervention techniques as well as the subsequent behavior.

The reliability of the variables may be affected by differences in individual wear times of the trackers. This was mitigated by setting a threshold of 1000 steps to consider the day valid. There is also a known tendency for Fitbit (and other trackers) to overestimate the amount of moderate to vigorous activity [13,14]. Furthermore, we used multiple observations for some volunteers to maximize the sample size. Although the rank-based nonparametric tests could not accommodate a random effect to account for the multiple observations, we checked an analysis, which averaged the observations per participant and found that the results and conclusions were the same.

The timing of the modules may have impacted the results. Although the order of visiting the modules was not technically restricted, the Goal session appeared earlier in the intervention flow and was visited by more participants than the Barriers session. It is thus possible that participants visiting the Barriers session were more committed to the intervention and, therefore, more likely to adhere to behavioral guidance as well. Further, engagement was only assessed based on log files and not by the quality of the action and coping plans done by the user, or the enactment of these.

### Future Work

As far as we know, no prior research to which we can directly compare these results exists. This shows the need for more research to examine the direct and immediate as well as longer-term effects of engaging with DBCI content on the enactment of the target behavior. Specifically, such work constitutes an important step toward identifying which behavior change techniques can have a differential impact on physical activity. This knowledge can, in turn, contribute to optimizing DBCIs in an adaptive and personalized way.

### Conclusions

A self-regulation–based intervention module addressing physical activity barriers induced a significant increase in active minutes, and the effect was stronger when the module was completed. Module completion was further associated with sustained increases, especially in fairly active and total active minutes.

## Appendix

Multimedia Appendix 1 Screenshots of the Goal module.

Multimedia Appendix 2 Screenshots of the Barriers module.

Multimedia Appendix 3 Additional analyses on background characteristics and activity metrics.

## Abbreviations

DBCI: digital behavior change intervention
TK: NoHoW Toolkit
