# Supplementary material for: Evaluation of the Immediate Effects of Web-Based Intervention Modules for Goals, Planning, and Coping Planning on Physical Activity: Secondary Analysis of a Randomized Controlled Trial on Weight Loss Maintenance
Source: J Med Internet Res. 2022 Apr 14;24(4):e35614. doi: 10.2196/35614 (PMC9052022; doi:10.2196/35614)
Supplement: Multimedia Appendix 1 [file jmir_v24i4e35614_app1.pdf]

8. Physical Activity Goal

Welcome back! Today you are invited **to set a new physical activity goal**. Whether you've been tracking you physical activity on your own or using the self-monitoring features in NoHoW, think about your typical physical activity levels, for example your daily steps, or time spent doing specific activities like cycling, swimming, dancing, or walking. **Use information about your current activity levels to set new goals**. If in a typical day you walk 6000 steps, try increasing it to 8000. If you've been swimming two days a week, consider increasing that to three days a week. **If you're feeling good about your current activity levels, then set a goal to maintain and keep up with what you've been doing**. Not sure where to start with exercise goals? Check out the most recent guidelines for physical activity to get some ideas. And use the tips below:

**Moderate-Intensity Physical Activity**<br><br>Your breathing and hearth rate increases but you can still have a conversation	**Vigorous-Intensity Physical Activity**<br><br>Fast paced heart rate, hard and fast breathing so that you can't maintain a conversation
Walking briskly (a 15-minute mile)	Jogging/running
Light yard work (raking/bagging leaves or using a lawn mower)	Swimming laps
Walk the dog	Most competitive sports (football, basketball, or soccer)
Actively playing with children	Jumping rope
Biking at a casual pace	Rollerblading/inline skating at a brisk pace

Four keys to goal setting:

- **Challenge yourself** - enough but not too much. You'll be bored with a goal that's too easy and get frustrated with one that's too hard.
- **Make it yours** - Setting a goal that is important and meaningful to you will make it more likely that you stick with it.
- **Make it realistic** - You may have a big, "ultimate" goal in mind. While it's great to have these kinds of goals, it takes awhile to get to them. **Give yourself a confidence boost and set some smaller goals that get you closer to your "big" goals, so you can see some progress and celebrate those successes.** For example, if you would like to run a 10K, but right now you are walking two days a week for about 30 minutes, you might consider starting with a run-walk plan, and build up to running 5, 10, 15, or 20 minutes without stopping.
- **Make it specific** - Some goals can be vague and difficult to measure. It is important to set goals that are as specific as possible with a detailed plan of action: "I will take a 30 minute walk during lunch Monday, Wednesday, and Friday" is more specific and more likely to be achieved than "I will exercise three days this week".

When setting your goals, answer these questions: What will I do? When will I do it? Where will I do it? With whom will I do it?

Let's Try This

Think about your current levels of physical activity. When it comes to your exercise behaviour, what do you want to do next? Maybe your goal is to keep doing what you're doing - to maintain. That's great! Maybe you want to challenge yourself to do a little bit more. When you are thinking about increasing your activity, remember to be realistic. Start by increasing just a bit. If you find that those smaller increases are too easy, then take it to the next level!

Here are some suggestions that will guide you in setting your own goals:

1. If you are usually getting in 8,000 steps a day in average, maybe your next goal is to try to get in 9500 steps a day. You can choose to spread this number of daily steps throughout the day, all days of the week or you may want to choose specific days and times when you will do more steps.

My current behavior: 8000 steps per day

My goal behavior (What): 9500 steps per day

How: bring walking shoes to work

When (which days): Monday to Friday

When (at what time): Lunch break

How long: 30 minutes

Where: Other outdoor space

With whom: Co-worker

2. If you are usually getting in 9,000 steps a day on average, maybe your next goal is to try to get in 11000 steps a day.

My current behavior: 9000 steps

My goal behavior (What): 11000 steps

How: By taking the dogs for walk after work, and jogging on weekends

When (which days): All days of the week

When (at what time): afternoon

How long: 30 minutes

Where: Neighbourhood

With whom: Alone

3. If you walk about 15 minutes per day, and you want to do extra 15 minutes 2 days per week. Remember that minutes of walking can also be converted into number of daily steps.

My current behavior: Walk 15 minutes daily

My goal behavior (What): Do extra 15 minutes walking 2 days per week

How: Walking to and/or from work

When (which days): Monday, Wednesday and Friday

When (at what time): Lunch break

How long: 30 minutes

Where: Other outdoor space

With whom: Alone

Now it's your turn! In the space below, indicate what your current level of activity is. Then, set your own goal. You can fill it for your daily steps, and also for other additional activities you may wish to pursue.

Steps per day	Other activity

My current behavior:

1000

My goal behavior:

What:

1000

How:

Hold control or command to choose multiple options

When (which days):

- Monday
- Tuesday
- Wednesday
- Thursday
- Friday
- Saturday
- Sunday

When (at what times):

- Morning
- Afternoon
- Evening
- Lunch/dinner break

How long:

1 hour

Where:

- Gym
- Other sports facility
- Home
- Neighbourhood
- Other outdoor space
- Other

With Whom:

- Partner or spouse
- Kids
- Other relative
- Friend
- Co-worker
- Exercise group
- Alone
- Other

Save goal

How useful did you find this session?

☆☆☆☆☆

You can add your notes here.

Save notes

Back to the map

Copyright © NoHoW 2016,2017,2018
