# Supplementary material for: Evaluation of the Immediate Effects of Web-Based Intervention Modules for Goals, Planning, and Coping Planning on Physical Activity: Secondary Analysis of a Randomized Controlled Trial on Weight Loss Maintenance
Source: J Med Internet Res. 2022 Apr 14;24(4):e35614. doi: 10.2196/35614 (PMC9052022; doi:10.2196/35614)
Supplement: Multimedia Appendix 2 [file jmir_v24i4e35614_app2.pdf]

NoHoW Back to the map Sign out
research in weight loss maintenance
What gets in the way... 14. Physical Activity Barriers

What Gets in the Way...

Welcome to the next NoHoW topic "What gets in the way...". Understanding the things that get in the way of achieving your goals is an important part of the weight management journey. **This topic has two sessions** - the first about physical activity and the second about healthy eating.

Each session starts with a testimonial. Sometimes hearing about the things other people have struggled with and how they have overcome those struggles can be really inspiring. After each testimonial, you'll have the opportunity to identify some of your own struggles when it comes to your goals.

The end of each session will support you in finding ways to deal with those difficulties that fit into your daily routine. Are you ready?

14. Physical Activity Barriers

Testimony

This is a story from George. You've met him before in previous session. George found that planning out what he would do to meet his physical activity goals was an important part of his weight loss journey. Here he talks about some of the challenges he has faced along the way.

George, 44 years old

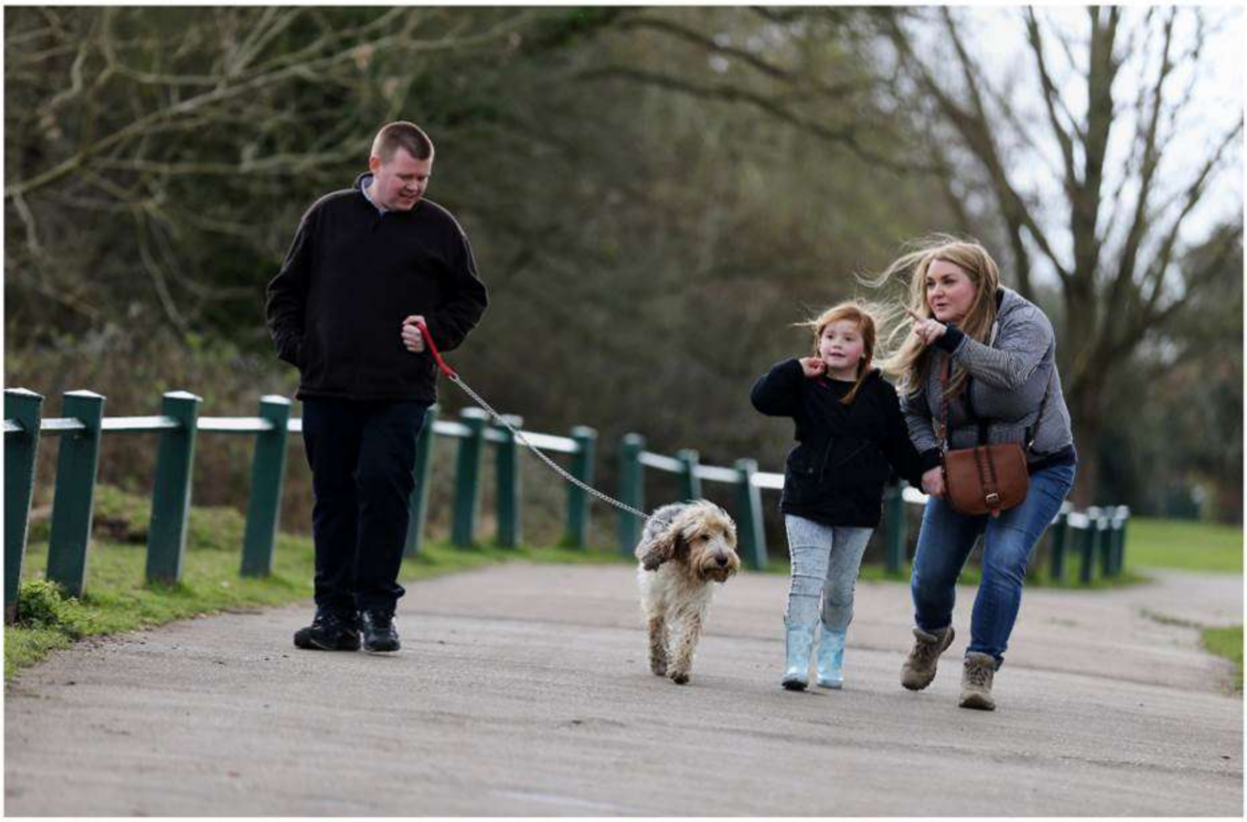

"As part of my weight loss journey, one of the main things I wanted to focus on was increasing my physical activity. Being active was always something I struggled with, so I started by setting a goal of walking at least 15 minutes every day. That got me started, but I wanted to challenge myself. So my next goal was to increase my daily activity to 20 minutes on weekends and adding in cycling when I could. I also started to up the intensity of my walking, so I was getting a good, fast walk in. I bought new athletic shoes and an activity tracker to help me keep an eye on my progress. For an added boost, I started parking farther away from the entrance to work and when I was out doing errands. I also took short walks after dinner on nights when I had to take the garbage out. Some days this was easy - especially early on. I was really excited about my new goals, and was already starting to see some improvements in how I was feeling - especially on the days when I got in some extra activity. Other times it was really hard. If traffic was bad and I felt like I was rushing to make it to work on time, parking farther away and taking the time to walk was the last thing on my mind. And some nights when I got home from work, I was tired - so going for a "bonus" walk after dinner seemed like just one more thing to do after an already-busy day. I knew from past experience that I had to figure out a way to work with these challenges - or being physically active would become a thing I used to do. So I tried out a few things. One strategy that worked really well for me was taking a short walk during my lunch break. I even marked them on my calendar - like an appointment - to stay on track. I found that going for a walk at lunch gave me so much energy to come back and finish the rest of my work day. And now, two of my friends at work have started walking with me. It's great to hang out with them on a walk, and knowing that they're counting on me being there gives me some added motivation to stick with it. There are still some days when it's hard to meet my goals, but now I feel more prepared to deal with those challenges. And every time I come up with a new strategy, it's like I'm creating a box of tools that I can use when I need it, and each tool fits different purposes, in this case, challenges."

Like George, at some point during your weight loss journey, **you've probably faced some challenges to being more physically active**. They may have been similar to the challenges George faced - lack of time or feeling tired, or you may have experienced other challenges - like lack of support from others.

You may have also noticed that some challenges were easier to tackle, while others took more effort. Sometimes challenges can be difficult to deal with because you don't yet have the skills or abilities to deal with them or you haven't yet found a way to deal with the challenge that really fits in your life. Other times, you might feel challenged with reaching a goal because your old way of doing things was working for you - maybe not having to carve out time for physical activity meant that you had more time to spend with friends or family.

To help you stick with your goals, it is useful to plan ahead for when challenges come up. It is also important to think of strategies that can fit into your life and to remind yourself of the reasons why improving your physical activity is important to you.

Challenge Yourself

Below is a list of barriers that are commonly reported by those trying to improve their physical activity. Select up to three that you have struggled with - or think you might struggle with - in pursuing your physical activity goal. Then rank them in order from the most challenging (number 1) to the last challenging (number 3).

Items	Your Selection
I feel too tired	1 I feel I don't have time
I am under stress or in a bad mood	
I feel discomfort or pain	
I don't enjoy it	
I don't feel confident or skilled	
I don't have enough support from others (e.g. family)	
I have to do it alone	
I have limited access to fitness facilities/equipment (e.g. financial, transport)	
The weather is bad (e.g. rain, too hot)	
I am traveling/on holidays	
[illegible]	

Now, think about your own strategies to cope with these challenges.

Here are some examples to help you:

- "If it's raining, then I will exercise at home."
- "If I am on holidays, then I will plan my exercise sessions in advance." (or I will look up gyms and other exercise locations that are available where I am traveling)
- "If I feel I don't have enough support from friends, then I will talk with them about the kinds of support I most need."
- "If I feel I don't have enough support from my family, then I will search for other sources of positive support."
- "If I feel discomfort or pain, then I will ask for advice from my general practitioner."
- "If I feel I don't have time, then I will do a shorter training session."
- "If I feel I don't have time, then I will take my exercise clothes with me to work so I can go straight to the gym from work."
- "If I don't have access to fitness facilities, then I will walk in the park near my house."
- "If I feel too tired after work, then I will walk at lunch or do my exercise session in the morning."
- "If I don't enjoy the gym classes, then I will choose an activity I can enjoy, for example walking in my neighborhood."

Coping Plan

Stating challenging situations as an "if" and providing the solution as a "then" is a powerful way to overcome challenges in maintaining your improved (or improving) physical activity. For the most challenging barrier to physical activity you selected above, write one solution you will try. Remember it is important that you choose solution that is realistic and will fit your preferences and daily routines.

If the first solution you have identified doesn't work the way you had hoped, try out a different one. These are always available in the "Steps Tile" of the toolkit, and you can change them at any time. You might want to come up with new solutions because you want to try something different to deal with the same or new barriers, or you have set new goals that may lead to new challenging situations. Remember, plan ahead how to overcome these challenges!

See You Soon

In this session you learned about how to develop strategies to deal with challenges to improve your physical activity.

This week, if challenges come up, try the strategies you've identified. It can work out really well, or not. The good news is that you can always try new strategies.

Think of strategies as tools that go into your weight management toolbox. Use the ones that work for you and add new ones when you need to.

How useful did you find this session?

☆☆☆☆☆

You can add your notes here.

Save notes

Back to the map

Copyright © NoHoW 2016,2017,2018
