# Supplementary material for: Evaluation of the Immediate Effects of Web-Based Intervention Modules for Goals, Planning, and Coping Planning on Physical Activity: Secondary Analysis of a Randomized Controlled Trial on Weight Loss Maintenance
Source: J Med Internet Res. 2022 Apr 14;24(4):e35614. doi: 10.2196/35614 (PMC9052022; doi:10.2196/35614)
Supplement: Multimedia Appendix 3 [file jmir_v24i4e35614_app3.docx]

# Multimedia Appendix 3 – Additional analyses on background characteristics and activity metrics

## Analysis on background characteristics

This analysis was conducted to investigate the differences in background characteristics between those who visited the modules and those who did not. Of the 811 participants for whom these modules were available 556/811 (68.6%) were women, their age was 44.09 (SD 11.54) years, and their average BMI was 29.49 (SD 5.31) kg/m^2^.

Gender, age and BMI were compared between visitors and non-visitors of the modules. For the Goal module, 348/498 (69.88%) of the visitors were women, and which did not significantly differ from the gender distribution in the whole population *Χ*^2^_1_ =1.1, *P*=.306. For the Barriers module, 295/406 (72.66%) were women, which was significantly more than in the whole population *Χ*^2^_1_ =6.4, *P*=.012. For both modules, the visitors were significantly older than non-visitors (see Tables 1 and 2). However, these results may apply to the Toolkit use in general and not only these modules.

Table 1. Comparison of demographics between Goal module visitors and non-visitors.

|  | Non-visitors | Visitors | *P* value^a^ |
| --- | --- | --- | --- |
| Age, mean (SD) | 40.97 (11.41) | 46.05 (11.19) | <.001 |
| BMI, mean (SD) | 29.75 (5.87) | 29.33 (4.92) | .134 |

^a^ T-Test

Table 2. Comparison of demographics between Barriers module visitors and non-visitors.

|  | Non-visitors | Visitors | *P* value^a^ |
| --- | --- | --- | --- |
| Age, mean (SD) | 41.62 (11.17) | 46.56 (11.38) | <.001 |
| BMI, mean (SD) | 29.53 (5.52) | 29.44 (5.10) | .404 |

^a^ T-Test

## Changes in physical activity metrics during the intervention

We compared Fitbit based activity metrics between the first and sixth month of the study (Table 3). The data were available (i.e., Fitbit had been worn), for 768/811 participants for the first month and 688/811 for the sixth month. The daily step count, activity energy expenditure, very active minutes, and distance were significantly lower during the sixth than the first month. Fairly active minutes were higher during the sixth month than the first. It is possible that receiving the Fitbits at start of Month 1 caused an intervention effect and increased the activity levels.

Table 3. Comparison of Fitbit-based activity metrics between the first and sixth month.

|  | Month 1, median (IQR) | Month 6, median (IQR) | *P* value*^a^* |
| --- | --- | --- | --- |
| Steps | 9743.83 (7583.60–12065.08) | 9133.45 (7267.23–11522.09) | <.001 |
| Energy (kcal) | 1281.83 (1017.14–1599.29) | 1243.79 (990.67–1536.13) | <.001 |
| Fairly active minutes (min) | 18.11 (11.10–29.28) | 19.81 (11.87–32.86) | <.001 |
| Very active minutes (min) | 23.70 (13.37–40.15) | 23.21 (12.58–39.83) | .038 |
| Total active minutes (min) | 45.18 (23.78–69.86) | 45.55 (27.92–72.41) | .426 |
| Distance (km) | 6.83 (5.31–8.52) | 6.44 (5.03–8.16) | <.001 |

^a^ Wilcoxon signed ranks test
